# Protective Effect of Boric Acid Against 3-NPA-Induced Ovarian Damage

**DOI:** 10.1007/s12011-025-04725-8

**Published:** 2025-06-27

**Authors:** Ömer Faruk Başer, Mahmut Karapehlivan, Abdulsamed Kükürt, Ayfer Yıldız Uysal, Esra Kafkas, Serpil Dağ

**Affiliations:** 1 Faculty of Medicine, Department of Medical Biochemistry, Kafkas University, Kars, Turkey; 2 Department of Biochemistry, Faculty of Veterinary Medicine, Kafkas University, Kars, Turkey; 3 Faculty of Veterinary Medicine, Department of Pathology, Kafkas University, Kars, Turkey

**Keywords:** Boric acid, 3-NPA, Oxidative stress, Apoptosis, Ovarian, Reproduction

## Abstract

In our study, the potential protective effects of boric acid (BA) against ovarian damage induced by 3-nitropropionic acid (3-NPA) were investigated. In an experiment conducted on female Wistar rats, the animals were divided into groups receiving control, 3-NPA, 3-NPA + BA, and BA only. 3-NPA triggers oxidative stress in the body by inducing excessive production of reactive oxygen species (ROS), leading to cellular damage and apoptosis. Experimental results showed that oxidative stress markers (MDA, GSH, and GSH-Px) increased in the group treated with 3-NPA, along with elevated inflammatory and apoptotic indicators (TNF-α, IL-6, NF-κB, caspase 3, and 9). Moreover, significant histopathological findings such as degeneration, necrosis, and oocyte loss in follicles were observed. Boric acid application significantly reduced these adverse effects, enhancing antioxidant defense and providing protective effects on ovarian tissue. In conclusion, it was observed that BA’s antioxidant and anti-apoptotic properties exhibited protective potential against 3-NPA-related ovarian damage, with a positive impact on reproductive health. The study suggests that BA might be used as a supportive agent in diseases of the female reproductive system.

## Introduction

The ovary, a pair of genital organs with a grayish-white color, is located immediately on the left and right sides of the uterus [1]. As the primary reproductive organ of female mammals, ovaries consist of structures called follicles. These follicles contain oocytes surrounded by two types of somatic cells—granulosa cells in the early stages of development and later theca cells. Granulosa and theca cells jointly play a role in synthesizing steroid sex hormones, known as estrogen and progesterone, which primarily regulate the female reproductive system in the body [2]. Throughout a female’s normal reproductive cycle, 300–500 mature oocytes undergo ovulation, and the vast majority of follicles (over 99%) are lost through an atresia process during folliculogenesis and in the primordial pool. The inability to replenish the oocyte pool makes this an irreversible process [3]. Nowadays, a decrease in fertility rates in both male and female individuals is observed due to xenobiotics accumulating in the body as a result of poor environmental conditions, pesticides applied to food, chemicals used in the food industry, and various toxic exposures [2, 4]. Due to ovarian dysfunction, along with infertility caused by estrogen deficiency, there are adverse effects on life quality, bone function, and cardiovascular and neurological health [5].

It has been reported that an increase in the production or presence of oxidative species, along with a decrease in the activity of antioxidants and the antioxidant system, leads to oxidative stress caused by high levels of reactive oxygen species (ROS) [6]. Excessive production of ROS may occur due to various factors in living organisms (infections, chemicals, diseases, dietary habits, etc.). Specifically, the accumulation of high levels of ROS present in the circulatory system in the ovaries has been reported to cause detrimental effects on the function and physiology of ovarian follicles and lead to pathological changes in the female reproductive system [7]. Oxidative stress in the ovaries has been reported to cause apoptosis in granulosa cells in follicles, follicular atresia, and oocyte damage [8]. Antioxidants have been proposed to play a significant role in maintaining oxidative balance in living systems and protecting against the harmful effects of oxidative stress. Along with endogenous antioxidants, exogenous antioxidants have also been reported to play important roles as oxidative stress antagonists [6].

3-nitropropionic acid (P-nitropropionic acid, bovinosidin, 3-nitropropionic acid (3-NPA), also known as hippagenic acid, is a chemically pure substance, easily soluble in water, and appears as a yellow, crystalline solid. The mechanism of toxicity is suggested to involve striatal artery damage, dopaminergic toxicity, or possibly the heightened activity of glutamate transporters. The LD-50 doses of 3-nitropropionic acid for rats and mice are reported as 60/120 mg/kg [9, 10]. The toxicity caused by 3-NPA in rodents is categorized into three stages: the first stage is drowsiness, the second stage involves walking characterized by rolling movements and lack of coordination, and the third and final stage is marked by dominant chewing movements and either lateral or ventral recumbency. Death is most commonly reported to result from respiratory system failure [11]. The main mechanism of 3-NPA’s effect is thought to be the inhibition of SDH—a component of mitochondrial ETC and the enzyme responsible for the dehydrogenation of succinic acid to fumaric acid in the citric acid cycle—thereby disrupting energy production [12]. It has been reported that the inhibition of SDH and the beta-oxidation of fatty acids leads to a disruption in the essential continuous energy production required for the organism, resulting in oxidative stress that may cause follicular atresia and ovarian damage. Additionally, in a previous study, we reported that 3-NPA induces ovarian damage [4].

Boron (B) compounds, particularly boric acid (BA) and borax, are widely used in the industrial sector for the preparation of disinfectants and pharmaceuticals [13]. Once boron compounds are ingested through diet, they are broken down in the digestive system and converted into BA (boric acid), which then forms complexes with biomolecules such as glycolipids and glycoproteins through its hydroxyl groups [14, 15]. Boric acid (H_3_BO_3_) is a natural dietary nutrient and trace mineral essential for human health, found in fruits, vegetables, and various other foods. Studies investigating the pharmacokinetics of BA in both humans and animals have revealed that orally administered boric acid is rapidly absorbed and distributed in the body through passive diffusion in rats, rabbits, humans, and other animal species [16]. The physiological and biochemical effects of BA on humans and animals are explained via three hypotheses [15]. The first is its involvement in the activity of channels located in cell membranes [17]; the second is its role as a regulator of various biochemical reactions, particularly in enzymatic activities [18]; and the third is its potential ability to reduce oxidative stress by activating glutathione reserves to lower ROS levels [13]. Previous experimental studies have shown that BA can be used as an antioxidant [19, 20].

We investigated the biochemical parameters and histopathological effects of boric acid applied as a protective agent against ovarian damage induced by 3-NPA.

## Material and Methods

### Animals

The study was initiated after obtaining ethical approval from the Local Ethics Committee for Animal Experiments of Kafkas University (KAÜ-HADYEK/2023–052). A total of 38 female Wistar albino rats, aged 10–12 weeks and weighing 180–220 g, were obtained from the Kafkas University Laboratory Animal Application and Research Center. The experimental phases of the study were conducted at the same center. Vaginal cytology was performed to determine the estrous cycle periods of the rats used in the study. After cytological examination, rats in the proestrus phase were included in the study. At the conclusion of the study, subjects in the luteal phase were included in the evaluation. Under experimental conditions of 22 ± 2 °C temperature, 60–65% humidity, and 12 h of darkness and 12 h of light, the subjects were provided with standard commercial rat pellets (Bayramoğlu Yem, Erzurum, Turkey) and water ad libitum.

### Drugs

The 3-NPA (CAS:504–88-1) and boric acid (CAS:10,043–35-3) used in the study were obtained from Sigma-Aldrich. Boric acid and 3-NPA were dissolved in physiological serum and administered to the rats.

### Experimental Design

Thirty-eight (38) female Wistar rats were divided as eight in the control group and ten in each of the other groups. Control group: Only physiological serum was administered throughout the experimental procedure. 3-NPA group: A total daily dose of 12.5 mg/kg [21] of 3-NPA was divided into two equal doses of 6.25 mg/kg, administered 12 h apart in the morning and evening for 7 days. 3-NPA + BA group: 100 mg/kg BA [22] was administered for 8 days, and starting from the 2nd day, 6.25 mg/kg 3-NPA was administered twice a day, 12 h apart, totaling 12.5 mg/kg daily, for 7 days until the 8th day. BA group: 100 mg/kg BA was administered daily for 8 days. All chemicals were prepared by dissolving them in isotonic saline, and all injections were performed intraperitoneally (IP).

### Sampling and Processing of Specimens

Twenty-four hours after the last application, all the rats were anesthetized using ketamine (15 mg/kg/i.p)-xylazine (10 mg/kg/i.p), and blood samples were collected via cardiac puncture into heparinized tubes for plasma and empty tubes for whole blood. Then euthanasia was performed by cervical dislocation. Ovarian tissues of the rats were collected and preserved in 10% formaldehyde solution for histopathological examination. Blood samples were centrifuged at 3000 rpm for 10 min, and the plasma was separated into a separate Eppendorf tube and stored at − 86 °C until the day of analysis.

### Biochemical Analysis

Whole blood GSH analysis was carried out according to the method of Beutler [23]. In this method, all proteins devoid of sulfhydryl (-SH) groups are precipitated, and -SH groups form yellow-colored complexes with DTNB, which are measured spectrophotometrically at a wavelength of 412 nm. The obtained data were expressed in mg/dL.

Plasma MDA levels were measured spectrophotometrically according to the method of Yoshioka [24]. In this method, MDA reacts with TBA under low pH and heating conditions to form a red-pink color. This color arises from a chromogen formed by the combination of one MDA molecule with two TBA molecules and was measured spectrophotometrically at a wavelength of 535 nm. The obtained data were expressed in µmol/L.

The plasma levels of TNF-α, NF-κB, and AMH were obtained using commercial kits purchased from BT LAB (Zhejiang, China) and analyzed following the manufacturers’ procedures (E0764Ra, E0287Ra, and EA0083Ra). Similarly, IL-6 and caspase 3 and 9 levels were obtained using commercial kits purchased from Shanghai YL Biotech Co., Ltd. (Shanghai, China) and analyzed accordingly (YLA0031RA, YLA0017RA, and YLA0366RA).

### Statistical Method

The data obtained from biochemical analyses were analyzed by ANOVA using GraphPad Prism 10 software. Data are expressed as mean ± standard deviation. A *p*-value of < 0.05 was considered statistically significant.

Tissue samples stained by immunohistochemical methods were analyzed under a microscope by two independent pathologists. Considering the percentage and intensity of immunopositive cells, the percentage of cytoplasmic immunostaining was scored as 1 if between 0 and 25%, 2 if between 26 and 75%, and 3 if greater than 76%. The staining intensity was scored as none or weak: 1; strong: 2; and very strong: 3 [25]. The sum of both parameters was calculated for all subjects and divided by the number of animals in each group to determine the immunohistochemical score of the groups. These were statistically evaluated, and a significance level of *p* < 0.05 was considered. According to the Kolmogorov–Smirnov test, it was found that the groups did not show a normal distribution (*p* > 0.05). Since the groups were independent, the Kruskal–Wallis test was applied.

### Histopathological Method

#### Hematoxylin–Eosin Staining

For hematoxylin–eosin staining, Section. 4 µm thick were taken from paraffin blocks and deparaffinized by passing through two separate xylene series for 10 min each. They were then rehydrated by passing through an alcohol series from high concentration to low concentration. For nuclear staining, the sections were stained with Mayer’s hematoxylin for 10 min. Afterward, they were washed with tap water, dipped once into 1% acid alcohol, washed again with tap water, and then soaked in ammoniated water for 3 min to achieve a bright blue color. They were washed for 2 min with distilled water, dipped into 96% ethanol, and counterstained with eosin solution for 5 min for background staining. Finally, dehydration was performed by passing through an alcohol series from 70 to 100%, and the tissues were made transparent by treatment with two separate xylene solutions for 10 min each before being mounted with entellan. After the closure process, the prepared specimens were examined under a light microscope (Olympus BX53) and photographed using the Cell^P ^P program (Olympus Soft Imaging Solutions GmbH, 3, 4).

#### Immunohistochemical Staining

In the study, the avidin–biotin peroxidase method was applied as the immunohistochemical staining (IHC) technique. Sections underwent deparaffinization and rehydration processes, and then they were incubated in a 3% hydrogen peroxide solution (H_2_O_2_) for 20 min to block endogenous peroxidase activity. For antigen receptor retrieval, the microwave method was applied (citrate buffer solution pH 6, 800 watts, 10 min). To prevent nonspecific staining, non-immune serum (Thermo Scientific Histostain IHC Kit, HRP, broad spectrum, REF:TP-125-HL) was applied to the sections for 30 min. Subsequently, sections treated with antibodies diluted in PBS were incubated overnight in a refrigerator (4 °C). The sections were then incubated with biotinylated secondary antibody (Thermo Scientific Histostain IHC Kit, HRP, broad spectrum, REF:TP-125-HL) and peroxidase-conjugated streptavidin (Thermo Scientific Histostain IHC Kit, HRP, broad spectrum, REF:TP-125-HL) for 30 min at room temperature. Following this step, AEC solution (Thermo Scientific Fisher REF: TA-125-HA) was added as a chromogen, stained with Mayer’s hematoxylin, and covered with immuno-mount.

After the closure process, the prepared specimens were examined under a light microscope (Olympus BX53), and these were photographed using the Cell^P ^P program (Olympus Soft Imaging Solutions GmbH, 3, 4).

AMH and AMHR immunohistochemical staining were analyzed semi-quantitatively.

Staining intensity (3: strong, 2: moderate, 1: weak, and 0: no expression) (Table 1) [26].

**Table 1:** AMH and AMHR expression grading according to the IHC staining method

Staining and expression intensity	Degree
Strong staining and expression	3
Moderate staining and expression	2
Weak staining and expression	1
No staining and expression	0

## Results

### Biochemical Results

#### MDA Levels

**Fig. 1:**
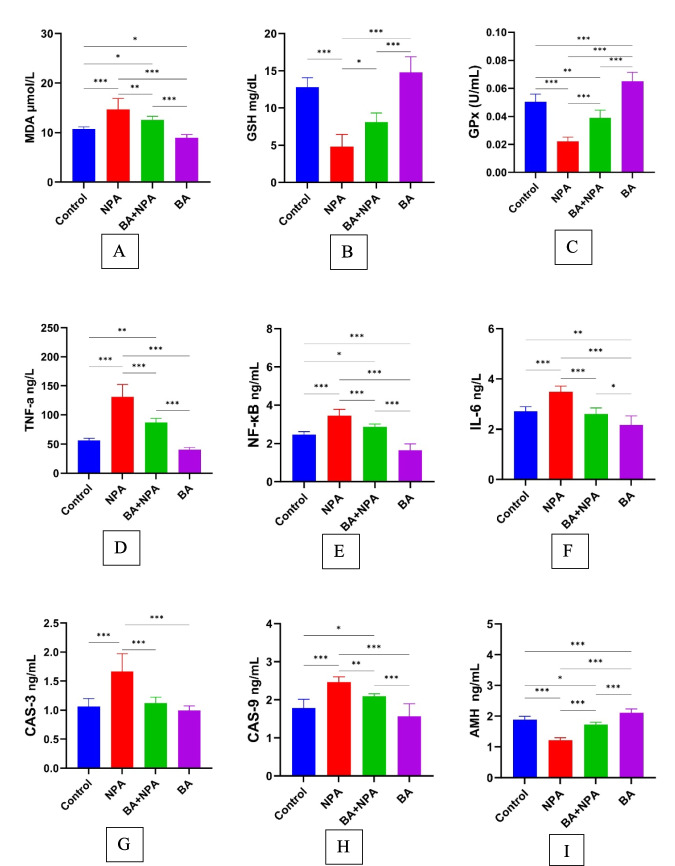
Biochemical results of all experimental groups. *presents *p* < 0.05; **presents *p* < 0.01, and ***presents *p* < 0.001

The changes in MDA levels between groups were displayed in Fig. 1A, based on the analyses conducted on plasma samples obtained at the end of the study. According to the data obtained, the group with the lowest MDA activity was identified as the BA group, while the group with the highest MDA activity was identified as the NPA group. Compared to the control group, significant increases were observed between the NPA and BA + NPA groups (*p* < 0.001, *p* < 0.05). Significant increases were also identified in the control (*p* < 0.05), NPA (*p* < 0.001), and BA + NPA (*p* < 0.001) groups compared to the BA group.

#### GSH Levels

According to the analysis of whole blood samples obtained at the end of the study, the variation in GSH levels among the groups is shown in Fig. 1B. Based on the data obtained, the group with the highest GSH level was the BA group, while the group with the lowest level was the NPA group. A significant decrease (*p* < 0.001) was observed in the NPA group compared to the control group. Compared to the BA group, a significant decrease was found in the NPA (*p* < 0.001) and B + NPA (*p* < 0.001) groups.

#### GSH-Px Levels

According to the analysis of plasma samples obtained at the end of the study, the variation in GSH-Px levels among the groups is shown in Fig. 1C. It was determined that the GSH-Px level was highest in the BA group and lowest in the NPA group. Significant differences were observed in the NPA (*p* < 0.001), B + NPA (*p* < 0.01), and BA (*p* < 0.001) groups when compared to the control group. Significant differences were also detected between the NPA (*p* < 0.001) and BA + NPA (*p* < 0.001) groups compared to the BA group.

#### TNF-α Levels

According to the analysis of plasma samples obtained at the end of the study, the variation in TNF-α levels among the groups is shown in Fig. 1D. Based on the data obtained, the group with the highest TNF-α levels was the NPA group, while the group with the lowest levels was the BA group. Significant differences were observed in the NPA (*p* < 0.001) and BA + NPA (*p* < 0.01) groups compared to the control group. Significant differences were also detected in the NPA (*p* < 0.001) and B + NPA (*p* < 0.001) groups compared to the BA group.

#### NF-κB Levels

According to the analysis of plasma samples obtained at the end of the study, the variation in NF-κB levels among the groups is shown in Fig. 1E. Based on the data obtained, the group with the highest NF-κB levels was the NPA group, while the group with the lowest levels was the BA group. Significant differences were identified among the NPA (*p* < 0.001), BA + NPA (*p* < 0.5), and BA (*p* < 0.001) groups compared to the control group. Compared to the BA group, significant differences were observed in the control, NPA, and B + NPA groups (*p* < 0.001).

#### IL-6 Levels

According to the analysis of plasma samples obtained at the end of the study, the variation in IL-6 levels among the groups is shown in Fig. 1F. Based on the data obtained, the IL-6 level was found to be high in the NPA group, while the group with the lowest IL-6 level was determined to be the BA group. Significant differences were observed in the NPA group (*p* < 0.001) and the BA group (*p* < 0.01) when compared to the control group. Compared to the BA group, significant differences were detected in the control group (*p* < 0.01), the NPA group (*p* < 0.001), and the B + NPA group (*p* < 0.05).

#### Caspase-3 Levels

According to the analysis of plasma samples obtained at the end of the study, the variation in caspase-3 levels among the groups is shown in Fig. 1G. Based on the data obtained, the highest caspase-3 level was observed in the NPA group, while the lowest caspase-3 level was found in the BA group. A significant increase (*p* < 0.001) was identified in the NPA group compared to the control group, and a significant increase (*p* < 0.001) was noted in the NPA group compared to the BA group.

#### Caspase-9 Levels

According to the analysis of plasma samples obtained at the end of the study, the variation in caspase-9 levels among the groups is shown in Fig. 1H. Based on the data obtained, caspase-9 levels were found to be the highest in the NPA group, while the lowest levels were observed in the BA group. Significant increases were identified in the NPA (*p* < 0.001) and BA + NPA (*p* < 0.05) groups compared to the control group. Significant increases (*p* < 0.001) were detected in the NPA and BA + NPA groups compared to the BA group.

#### AMH Levels

According to the analysis of plasma samples obtained at the end of the study, the changes in AMH levels among groups are shown in Fig. 1I. Based on the data obtained, it was determined that the BA group had the highest AMH levels, while the NPA group had the lowest AMH levels. Compared to the control group, significant differences were found in the NPA and BA groups (*p* < 0.001) and the BA + NPA group (*p* < 0.05). Additionally, significant differences were observed between the NPA and BA + NPA groups compared to the BA group (*p* < 0.001).

### Histopathological Results

#### Macroscopic Findings

No significant changes were observed during the macroscopic examination of ovarian tissues obtained from the sacrificed subjects at the end of the experiment.

#### Histopathological Evaluation Results

**Fig. 2:**
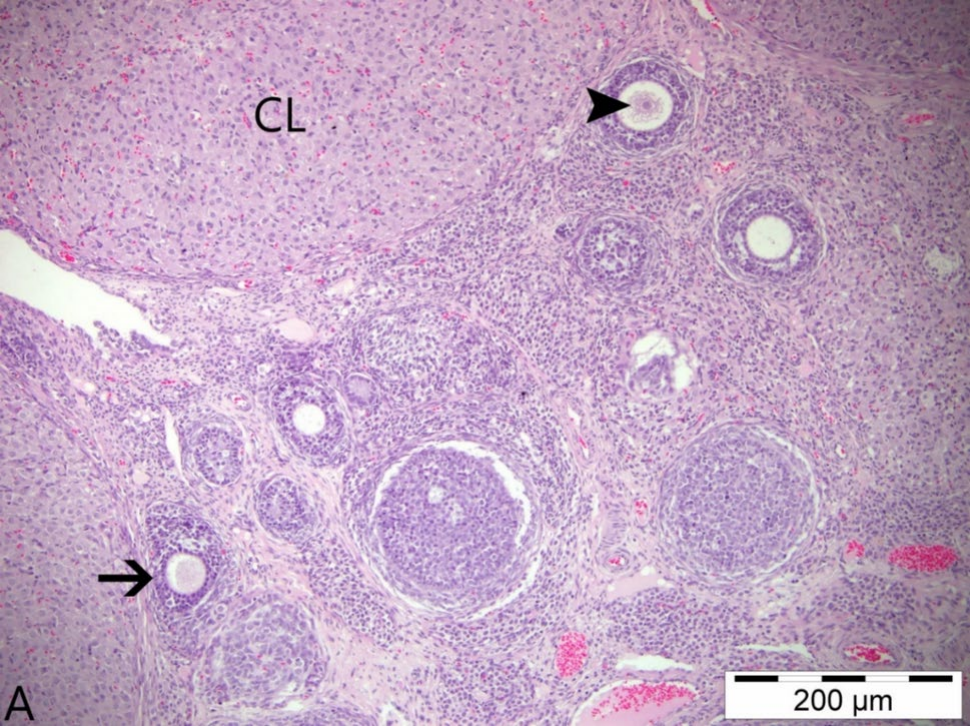
Ovarian tissue of the control group: normal histological structure; CL, corpus luteum; arrowhead: oocyte and preantral follicle; arrow: small antral follicle; H&E, 200 µm

**Fig. 3:**
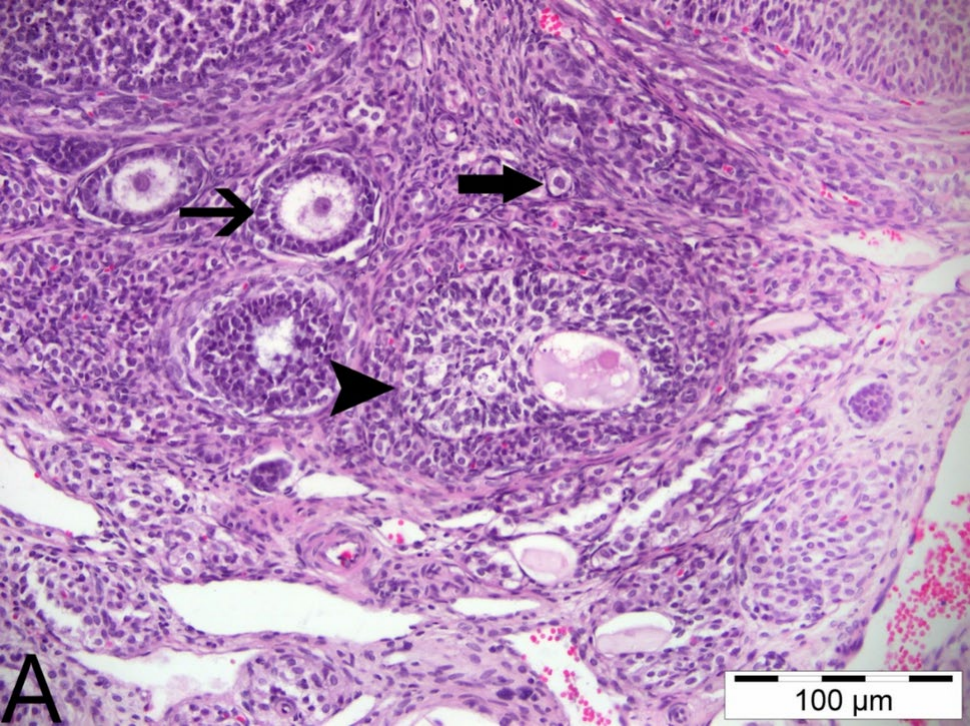
Ovarian tissue of the boric acid group: normal histological structure; arrowhead: antral follicle; thin arrow: preantral follicle (secondary follicle); thick arrow: primordial follicle; H&E, 100 µm

**Fig. 4:**
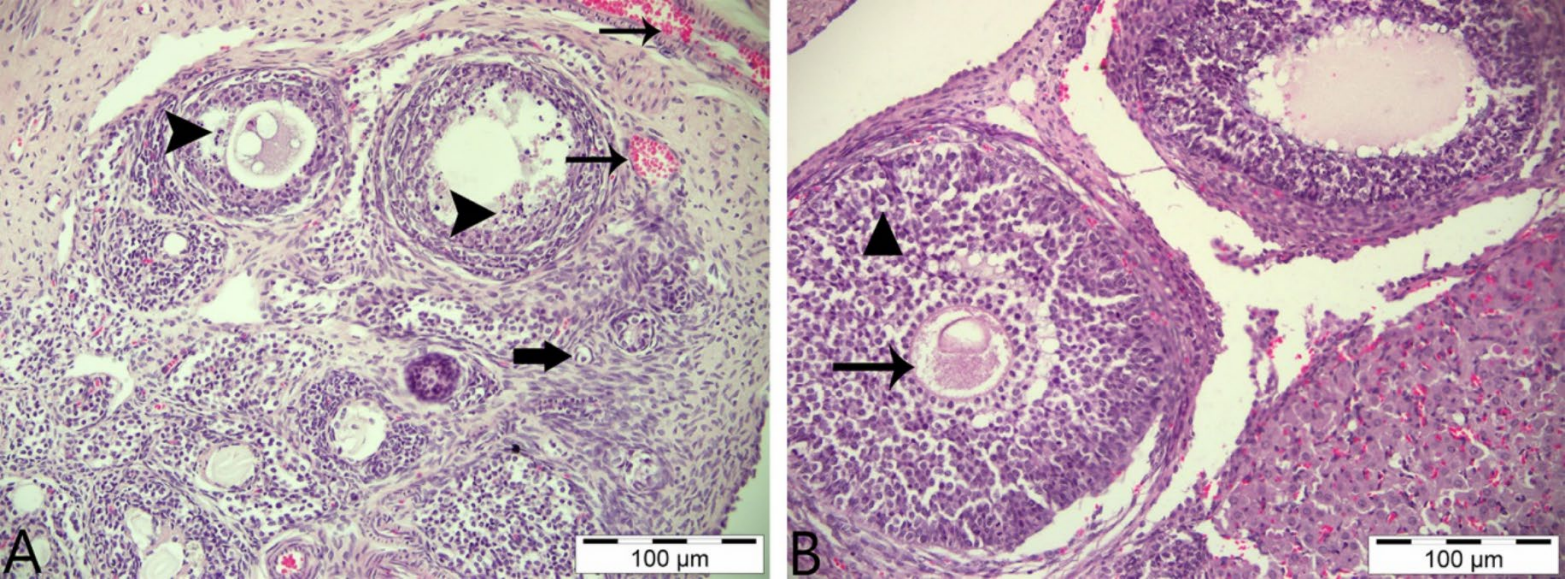
Ovarian tissue of the 3-NPA group: **A** arrowheads: antral follicles, necrosis in follicle granulosa cells, degeneration; thick arrow: primordial follicle; thin arrow: hyperemia in ovarian capillary vessels. **B** triangle: degeneration in granulosa cells; thin arrow: oocyte loss; H&E, 100 µm

**Fig. 5:**
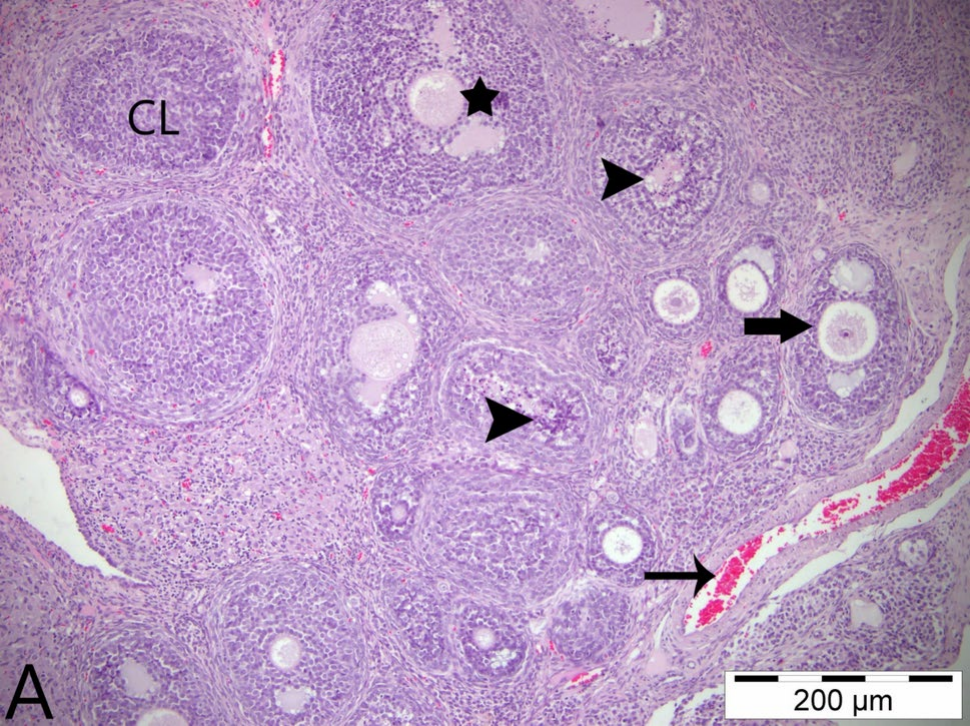
Ovarian tissue of the 3-NPA + boric group: CL, corpus luteum; thick arrow: antral follicle; arrowhead: granulosa cell necrosis and degeneration in the center of the follicle; star: oocyte loss; thin arrow: vascular congestion in ovarian tissue; H&E, 100 µm

In the histopathological examinations conducted, ovarian tissues from all groups were analyzed in terms of follicles, atretic follicles at different stages of development, degenerative and necrotic changes, and the presence of ovum. As a result of the examinations, no pathological findings were detected in the ovarian tissues obtained from animals in the control group and the group that was only given boric acid (Figs. 2 and 3). When comparing ovarian tissues from animals in the 3-NPA group with the other two groups, it was observed that subjects in the 3-NPA group displayed oocyte loss in developing antral follicles, vacuolar degeneration in granulosa cells of the follicles, and nuclear fragmentation due to necrotic changes in granulosa cells. Furthermore, disruptions and losses in the arrangement of corona radiata cells and hyperemia in vascular structures of the ovarian tissue were identified (Fig. 4). In animals given 3-NPA + boric acid, fewer follicles were observed with degenerative and necrotic changes, and the regression of lesions was identified compared to the group given only 3-NPA (Fig. 5).

The groups were compared in terms of the number of preantral, antral, and atretic follicles present in ovarian tissue. For this, three fields were selected in each ovarian tissue at × 20 magnification, and the preantral, antral, and atretic follicles in these fields were counted. The averages were calculated, and the number of preantral, antral, and atretic follicles for each group was determined (Table 2) [26]. Upon evaluation, the control group had the highest number of preantral follicles, while the 3-NPA group had the lowest. Similarly, mean antral follicle numbers were highest in the control group and lowest in the 3-NPA group. It was observed that the boric acid and 3-NPA + boric acid groups had averages close to the control group. In comparisons regarding atretic follicles, the highest average was found in the 3-NPA group, while the control group had the lowest.

**Table 2:** The number of preantral, antral, and atretic follicles of the groups

Groups	Preantral	Antral	Atretic follicle count
Control group	5.2	7.6	0.5
Boric acid group	5.8	7.1	0.8
3-Npa group	3.8	6.5	3.3
3-Npa + boric acid group	4.5	4.6	1

### Immunohistochemical Results

**Fig. 6:**
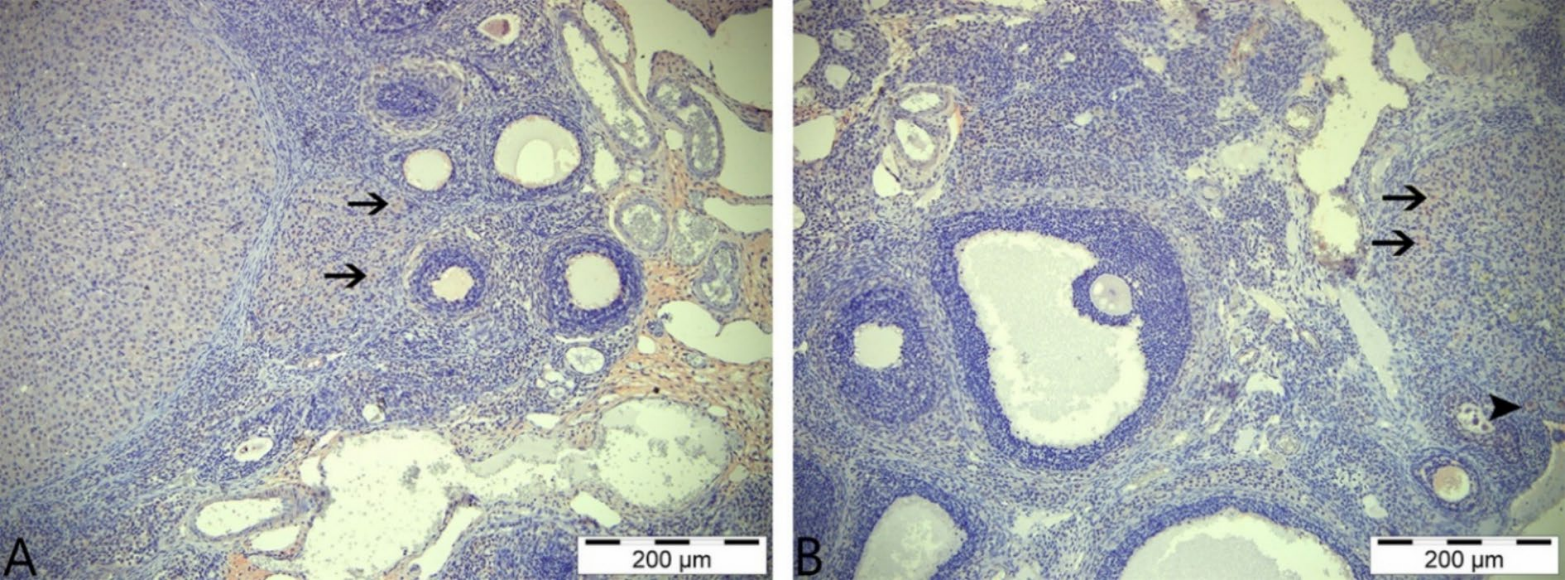
AMH immunohistochemical staining. **A** Control group—arrows: weakly immunopositive reaction in luteal cells. **B** Boric acid group—arrows: weakly immunopositive reaction in luteal cells; asterisk: positive immunoreactivity in primordial follicles; IHC, 200 µm

**Fig. 7:**
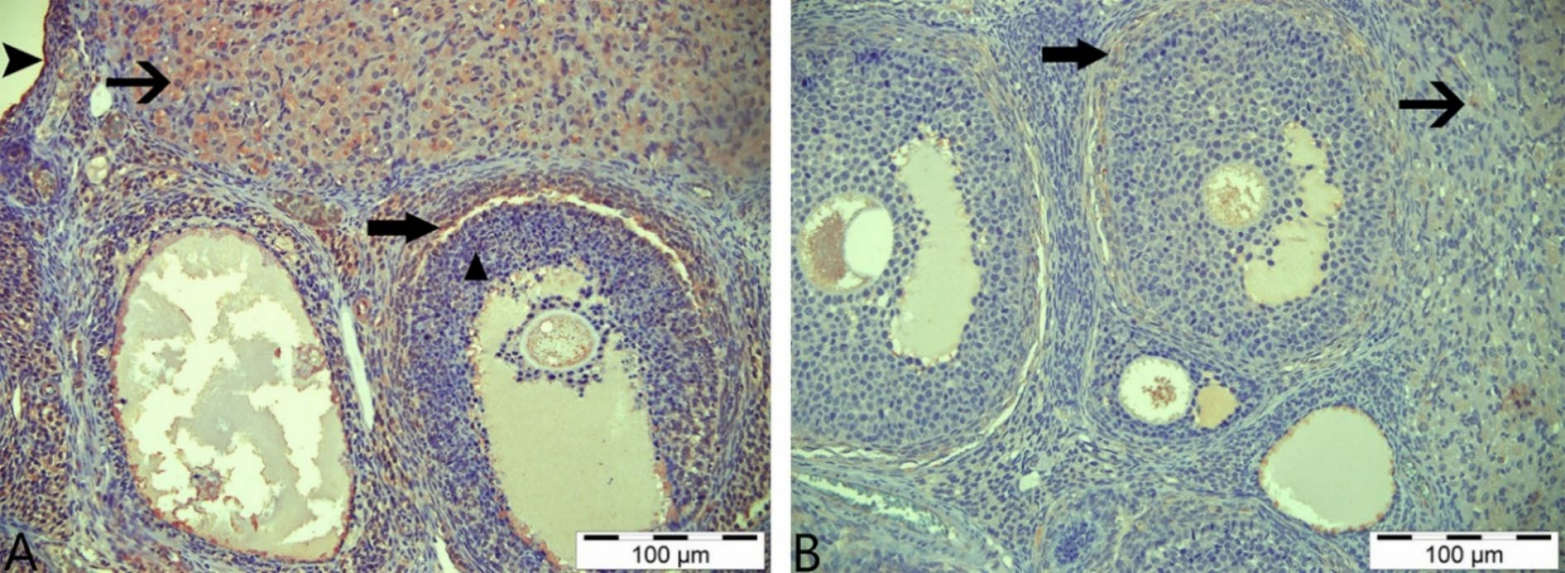
AMH immunohistochemical staining. **A** 3-NPA group—thin arrow: immunopositive reactions in luteal cells; thick arrow: positive immunoreactions in theca interna cells; triangle: positive immunoreactivity in granulosa cells; arrowhead: positive immunoreactions in str. germinativum cells. **B** 3-NPA + boric acid group; thin arrow: immunopositive reaction in luteal cells; thick arrow: positive immunoreactivity in theca interna cells; IHC, 200 µm

In immunohistochemical staining performed using the AMH primary antibody, very weak immunopositive reactions were detected in the luteal cells of the control and boric acid-only groups. In addition, very weak immunoreactions were observed in some primordial follicles in the boric acid group (Fig. 6A, B). Immunohistochemical staining of tissue samples from the group given 3-NPA revealed that immunopositive reactions were intracytoplasmic, especially in the theca interna cells surrounding the atretic follicle, granulosa and luteal cells, primordial follicles, and the cells in the stratum germinativum layer (Fig. 7A). In the staining performed in the group given 3-NPA + boric acid, a decrease in the intensity of reactions compared to the group given only 3-NPA was observed, with positive reactions detected in luteal cells and theca interna cells (Fig. 7B).

**Fig. 8:**
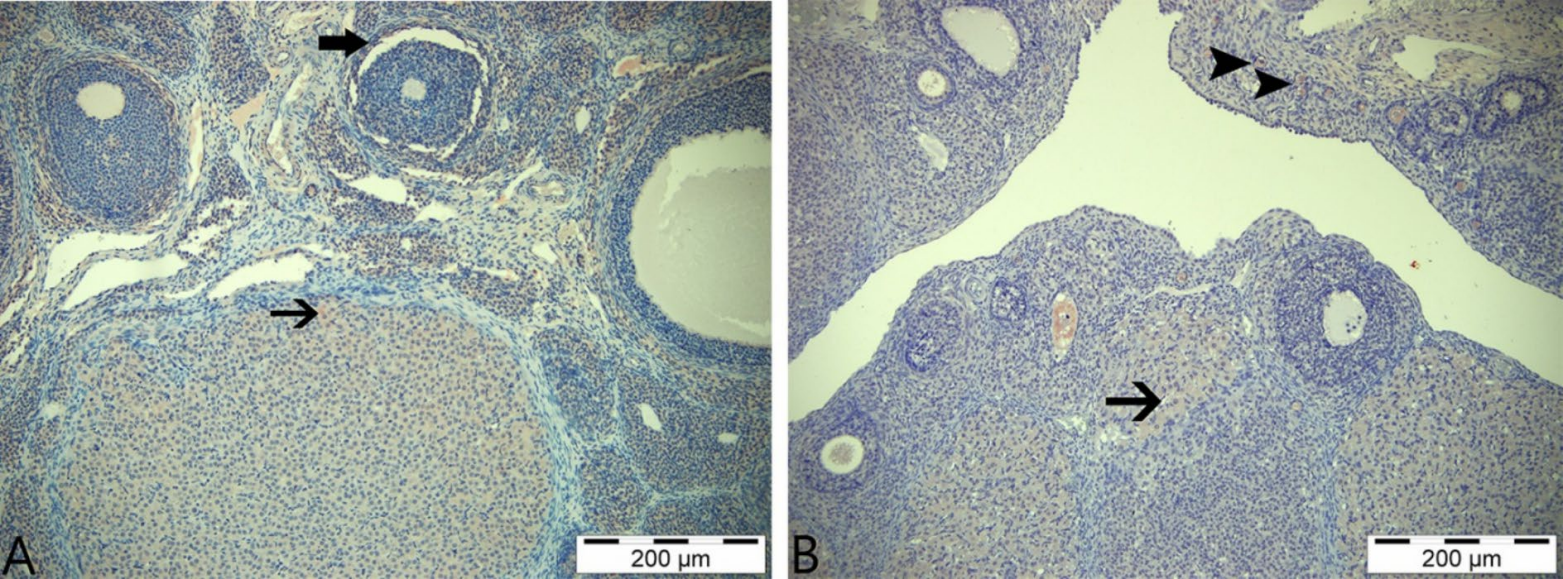
AMHR immunohistochemical stains. **A** Control group—thin arrow: positive immunoreaction in corpus luteum cells; thick arrow: positive immunoreactivity in theca interna cells. **B** Boric acid group—thin arrow: immunopositive reaction in luteal cells; arrowhead: positive reactions in primordial cells; IHC, 200 µm

**Fig. 9:**
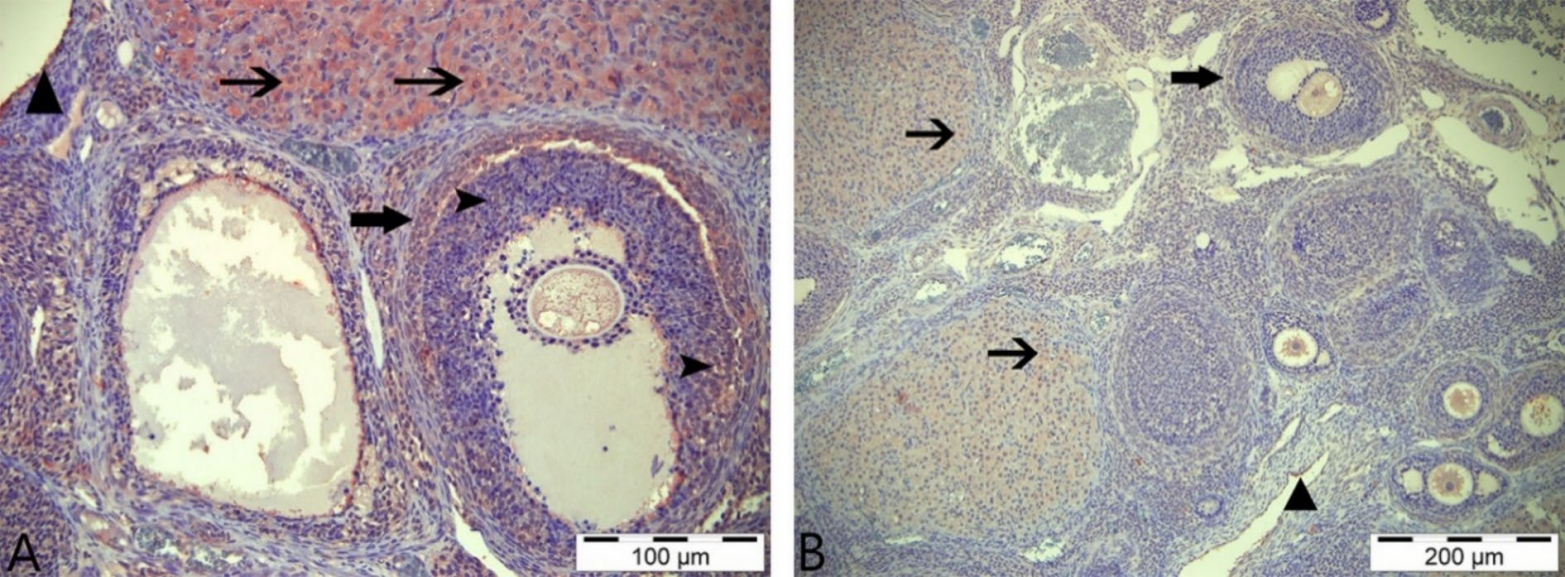
AMHR immunohistochemical staining. **A** 3-NPA group—thin arrows: immunopositive reactions in luteal cells; thick arrow: positive reaction in theca interna cells; arrowhead: positive immunoreactivity in granulosa cells; triangle: positive reaction in stratum germinativum cells. **B** 3-NPA + boric acid group—thin arrow: immunopositive reactions in luteal cells; thick arrow: immunopositive reactions in theca interna cells; triangle: positive reaction in stratum germinativum cells; IHC, 200 µm

In staining performed with the AMHR primary antibody in the control group, immunopositive reactions were found in luteal cells and theca interna cells (Fig. 8A). Immunohistochemical staining of the boric acid group revealed positive immunoreactions in luteal cells and primordial follicles (Fig. 8B). Immunohistochemical staining of the group given 3-NPA identified, similar to that with the AMH primary antibody, positive reactions in granulosa cells at the center of the follicle, in the theca interna cells surrounding the follicle, and in luteal and primordial follicles as well as stratum germinativum cells (Fig. 9A). In the group given 3-NPA + boric acid, positive reactions similar to those of the 3-NPA group were observed in luteal cells, theca interna cells, and str. germinativum cells with lower intensity (Fig. 9B).

The findings revealed that AMHR immunopositive reactions were stronger and more intense compared to AMH immunopositive reactions. Furthermore, in the semiquantitative comparison among the groups, the group with the strongest staining property and intensity for AMH and AMHR immunopositive reactions was the 3-NPA group, while the lowest reactions were observed in the control and boric acid-only groups. In the immunohistochemical comparison among the groups, the 3-NPA group was represented as “3,” the 3-NPA + boric acid group as “2,” and the control and boric acid-only groups as “1.”

**Fig. 10:**
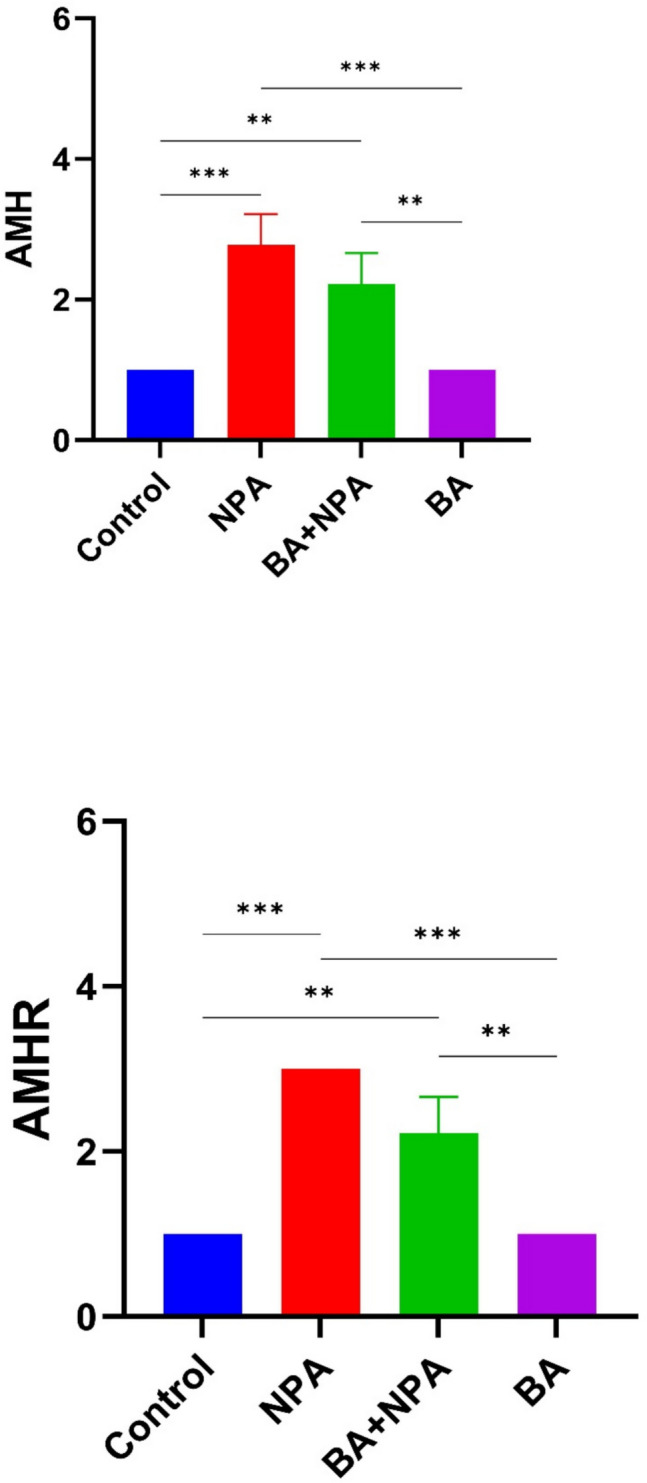
Statistical analyses of AMH and AMHR immunohistochemical stainings

Statistical analyses of AMH and AMHR immunohistochemical staining revealed (Fig. 10) a significant difference among the control, boric acid, 3-NPA, and boric acid + 3-NPA groups (*p* < 0.05), except between the control and boric acid groups, where no significant difference was observed (*p* > 0.05). Both AMH and AMHR immunohistochemical staining were significantly higher in the 3-NPA group compared to all other groups. Although boric acid administration reduced the effect induced by 3-NPA, immunoreactivity remained significantly higher than in the control and boric acid-only groups.

## Discussıon and Conclusion

Ovaries are defined as organs responsible for the secretion of sex hormones in females and the production of eggs necessary for reproduction of living organisms. The proper physiological functioning of the ovaries is very important for maintaining body homeostasis. ROS is reported to be formed as a result of the physiological metabolism of the ovaries, as in every system [27]. The balance between antioxidants and ROS in the organism determines the state of oxidative stress, and disruption of this balance in favor of ROS leads to problems in female reproductive systems such as ovulation, fertilization, oogenesis, and implantation. As a result, it is thought that these can cause physiological and pathological outcomes that may affect pregnancy in living beings [28]. The effects of oxidative stress arise due to the peroxidative damage of macromolecules and membranes of cells in living organisms and the impairment of metabolic activities in cell components. It is known that excessive oxidative stress in the body leads to organ and tissue pathologies [29]. Since the mechanisms of these physiological and pathological processes have not been fully elucidated, it is noted that externally supplied antioxidants might be a strategy to treat reproductive system diseases by reducing oxidants produced in the organism and regulating oxidative stress, and that further advanced studies are needed regarding oxidative stress-antioxidant support [30].

3-NPA has been proven in in vivo studies to cause oxidative stress by generating excessive amounts of free radicals and to trigger cell apoptosis [28]. Many studies have reported that 3-NPA leads to mitochondrial dysfunction by blocking mitochondrial complex II and SDH, and as a result, it induces oxidative stress and blocks ATP production [31, 32]. For this reason, 3-NPA was used in this study to induce ovarian damage and oxidative stress, and these were determined histopathologically and biochemically.

The element known as boron, a nonmetal, can be found in water, soil, and rocks. Globally, boron and its compounds are used in a wide variety of industries. The main types of boron to which humans are typically exposed are boric acid or borates. Most of the boron compounds consumed are hydrolyzed to boric acid and then circulate in body fluids such as blood and urine [33]. Boron-containing substances have a variety of biological effects, including antibacterial [34], antiviral [35], antifungal [36], anticarcinogenic [37], anti-invasive, anti-angiogenic [19], and anti-inflammatory [38]. In daily life, humans are most commonly exposed to boron orally. It is known that the oral route is predominant for boron exposure in people’s daily lives. Humans are generally exposed to boron through food and beverages, mostly in the form of borate or boric acid. Therefore, every individual is exposed to a certain amount of boron in daily life. Kar et al. [22] suggested that BA (boric acid) exhibits antioxidant properties by breaking the protons in oxidant molecules or by absorbing free radicals into its own structure.

The cells’ ability to regenerate GSH (either through resynthesis of GSH or reduction of GSSG) reflects the cell’s capacity to reduce oxidative stress [39]. In our study, it was found that 3-NPA administration reduced the capacity to produce GSH in the groups and weakened ROS-induced cellular defense. It was determined that BA given as a protective agent increased the capacity to regenerate GSH compared to the groups administered 3-NPA. In addition, we think that the GSH concentration may have been depleted in combating the excessive ROS caused by 3-NPA or that the blockage in ATP synthesis caused by 3-NPA may have had a negative effect on GSH synthesis.

GSH-Px is a selenoenzyme that inactivates H_2_O_2_ and a wide variety of lipid hydroperoxides [28]. In our study, it was determined that GSH-Px activity decreased in the groups treated with 3-NPA compared to the other groups, while only the group treated with BA showed an increase in activity. We think that the main reason for this increase and decrease is the excessive amount of ROS caused by 3-NPA and the organism’s struggle against the increased ROS. In a study by Hao and colleagues, sodium arsenite was administered to rats, and the decrease in GSH-Px activity in ovarian tissue supports our findings [40]. Another study investigated the protective effect of boric acid on liver tissue by administering cyclophosphamide to subjects to induce liver damage. Upon examining the results of our study, we found that BA increased GSH-Px activity [41]. We believe that BA enhances the organism’s defense against free radicals by boosting the activities of antioxidant enzymes.

MDA, one of the lipid peroxidation markers, appears as a peroxidation product of polyunsaturated fatty acids [42]. MDA is one of the most effective biochemical markers for lipid peroxidation occurring in the cell membrane, the production of RIS, and antioxidant consumption [43]. In our study, it was found that lipid peroxidation increased in the groups treated with 3-NPA and that BA, administered as an antioxidant, reduced this effect. We think that the reason 3-NPA increases lipid peroxidation is, as mentioned, due to blocking SDH and decreased ATP synthesis. The excessive production of free radicals and their binding to DNA increase pro-apoptotic signals [44]. With this increased signal, cytochrome c in the mitochondria passes into the cytosol, thereby activating caspase-3 and promoting cell death [45]. In our study, caspase-3 and 9 activities were found to be higher in the group treated with 3-NPA, while this activity decreased in the group given BA as an antioxidant. In the NPA-treated groups, we think the increase in caspase-3 and 9 is due to the increased release of ROS from mitochondria and the damage to mitochondrial DNA [4].

It is known that TNF-α has two different effects, the first being the regulation of the immune response and the support of defense mechanisms by regulating the functions of effector cells, and the second being its significant role in the inflammatory process [46]. In our study, it was observed that TNF-α levels were higher in the NPA-treated groups, while they were lower in the BA-treated groups. We think that the increase in TNF-α is due to the increase in ROS caused by 3-NPA, while the decrease in the BA groups results from its antioxidant properties. The finding that BA administered as a protective agent in the sepsis model conducted by Cao and colleagues reduced TNF-α levels supports our findings [46]. Similarly, in a study by Başeğmez and colleagues, the decrease in TNF-α levels with increasing BA dose supports our results [47].

IL-6 is a multifunctional cytokine involved in host defense and primarily regulates the inflammatory response [48]. In one study, a diabetes mellitus (DM) model was established in subjects, and the effects of boron and its derivatives were investigated by administering three different boron compounds: boric acid (BA), cyclohexylboronic acid (CHB), and phenylboronic acid (PBA). Analysis of the data at the end of the study showed that the group with the lowest IL-6 level was the BA group [49]. In our study, while the IL-6 level in the 3-NPA group increased compared to all other groups, BA administration was found to reduce the IL-6 level. We believe that the increase in IL-6 is due to elevated ROS caused by 3-NPA and the resulting damage to ovarian tissue, whereas BA decreases IL-6 levels by preventing 3-NPA-induced damage and reducing oxidative stress, thereby modulating the inflammatory process.

NF-κB is a potent proinflammatory nuclear transcription factor and is regarded as a central mediator of immune and inflammatory responses [50]. In our study, we observed a marked increase in NF-κB levels in the groups treated with 3-NPA, while a significant decrease was noted in the BA group. We suggest that the elevation in the 3-NPA group is associated with the damage caused by 3-NPA to ovarian tissue and the mitochondrial injury it induces, whereas the reduction in the BA + 3-NPA group is due to BA’s ability to diminish the oxidative stress triggered by 3-NPA. The decrease in NF-κB levels observed in the ovarian tissue ischemia/reperfusion model by Karaman and colleagues further supports the antioxidant properties of BA [51].

AMH is synthesized in the ovaries in females and regulates folliculogenesis. In clinical practice, AMH levels are considered a marker of the growing follicle pool, which in turn is related to the size of the primordial follicle pool. In short, AMH levels are accepted as a reliable indicator of ovarian reserve [52]. According to a previous study, a decrease in AMH levels after ovarian damage may result from ovarian follicle atrophy, an increase in the production of free oxygen radicals, and the accumulation of activateds [53]. As a result of our study, it was determined that the application of 3-NPA significantly reduced AMH levels, whereas the administered BA significantly increased AMH levels. It is supported by studies that 3-NPA treatment prolongs the diestrus phase, shortens the estrus phase, and reduces the number of primordial follicles, ovarian index, AMH levels, and fertility [54]. We think that the BA supplementation increases AMH levels both by reducing oxidative stress and by preventing tissue destruction. However, it should not be overlooked that AMH levels may also be affected by individual factors such as age and weight.

If antioxidants cannot control ROS, ovarian damage occurs [55]. Oxidative stress is characterized by a serious imbalance between free radical production and antioxidant defense mechanisms, leading to tissue damage [56]. In a study by Karaman and colleagues, ovarian damage was induced with the I/R model, and BA was administered orally, intraperitoneally, and locally at a dose of 15 mg/kg. When the obtained data were examined, a decrease in MDA levels, an increase in AMH quantity, and a decrease in NF-κB levels were reported in all groups that received BA [51]. The data obtained in this study support the results of our study, but we think that the higher protective efficacy determined was due to the high dose of boric acid we applied in the study. In another study conducted in 2022, ovarian damage was induced with I/R, and the effect of BA was investigated. When the data obtained in this study were examined, it was reported that, in groups given BA, there was a decrease in MDA and IL-6 levels and an increase in GSH levels, consistent with ours [15]. In a study conducted on the human ovarian cancer cell line MDAH-2774, different doses of BA were applied, and the data showed that oxidative stress levels increased in the group thated BA [57]. We believe this discrepancy with our study is primarily due to the fact that the organism is a whole, working in harmony with all other systems to increase survival. In addition, the dose amounts administered in cell culture are very minimal, and furthermore, the defense systems of humans and rodents may also affect these results. In a study conducted in 2025, high-fructose corn syrup was administered to pregnant subjects to induce damage in the uterus, placenta, and fetal tissues, and as a result, inflammation was reported to increase. The study found that high fructose intake led primarily to increased levels of inflammatory markers such as MDA, IL-6, and TNF-α, while GSH levels, an indicator of oxidative stress, decreased. However, in the same study, it was reported that in groups given BA, the levels of MDA, IL-6, and TNF-α significantly decreased, while GSH levels increased. These findings support the potential protective role of BA in reducing inflammation and oxidative stress. These results are also in line with the data from our current study [58].

In conclusion, 3-nitropropionic acid (3-NPA) increases cellular oxidative stress by triggering the formation of reactive oxygen species. This compound causes DNA damage and, by inhibiting the mitochondrial electron transfer chain, directs cells towards apoptosis. In the ovaries, exposure to 3-NPA leads to cellular degradation and adversely affects follicle development. In contrast, boric acid (BA), due to its antioxidant properties, provides cellular protection. BA reduces oxidative damage by neutralizing free radicals and supports cell survival by activating antiapoptotic mechanisms. Additionally, it is supported by scientific studies that BA has a protective effect in ovarian tissue and positively influences follicular developments [51].

## Data Availability

No datasets were generated or analysed during the current study.
